# An HIV Prevention Protocol Reviewed at 15 National Sites: How do Ethics Committees Protect Communities?

**DOI:** 10.1525/jer.2008.3.2.77

**Published:** 2008-06

**Authors:** Bethany Griffin Deeds, Marné Castillo, Zephyr Beason, Shayna D. Cunningham, Jonathan M. Ellen, Ligia Peralta, Adolescent Trials Network for HIV/AIDS Interventions

**Affiliations:** University of Maryland Medical School; The Children's Hospital of Philadelphia; Stroger Hospital of Cook County; Johns Hopkins University; Johns Hopkins University; University of Maryland Medical School

**Keywords:** HIV, community, ethics committee, adolescent

## Abstract

To learn whether ethics committees reviewing community-based participatory research concentrate on the protection of communities, in addition to individual participants, data from 15 sites were analyzed. Eighty-two ethics committee concerns related to consent (35%), protocol procedures (49%), data collection (17%), and HIPAA (6%) were identified. Concerns generally involved individual level subject issues; only 17% were related to community issues. To improve community-level protections in research, the authors recommend that both ethics committee members and research staff receive education concerning protection and respect for communities, that a community member group be established to advise researchers throughout the planning and implementation of community-level studies and that local ethics committee boards include members with community-level experience.

Research In Public Health And Medicine often includes multiple levels of investigation (e.g., institution-level data from communities, neighborhoods, or schools) in addition to individual-level data (IOM, 2002; Weijer, 1999). Research that places a high value on developing a collaborative process between researchers and community participants, such as community-based participatory research (CBPR) and participatory action research (PAR), (Khanlu & Peter, 2005; Marshall & Rotini, 2001) is gaining wide support for its potential to reduce health disparities (Olshansky et al., 2005), and promote health and prevent disease (Linnan et al., 2005; DHHS, 2005). Such research often includes multiple levels of investigation. Several studies have explored the special ethical challenges of involving the community as an integral component of research in CBPR and PAR (Brugge & Kole, 2003; Minkler, 2004; Minkler et al., 2002) and the process of soliciting community consultations during the research process (Duggan, Jaruis, Derauf, Aligne, & Kaczorowski, 2005; AAP, 2004; Sixsmith, Boneham, & Goldrig, 2003; Quinn, 2004). In addition, a limited number of studies have evaluated the protection of community rights in the context of genetics research (Weijer, 2000). There do not exist, however, any regulations in the U. S. Federal Policy for the Protection of Human Subjects on how best to protect the *rights of communities* when they are the subject of investigation. Thus, researchers must rely somehow on human subject protection procedures originally designed to protect individuals participating in biomedical research for guidance.

Since the individual no longer is the sole focus of public health and medical research, it is important to assess how essential human subject protections originally designed for individuals participating in biomedical research can be applied to research where *the community* is a unit of focus. The objectives of this paper are to describe the following:

1. The study design, human subjects protection features, and key community protection concepts of a CBPR protocol where the community is both a unit of investigation itself (direct focus), and is impacted by individual-level research (indirect focus).
2. Individual- and community-related concerns raised by local ethics committees during the review process of this research protocol.
3. The best practices developed at the research sites to effectively resolve these concerns.

## Background

### Connect to Protect^®^

The Adolescent Medicine Trials Network for HIV/AIDS Interventions (ATN) is a multi-center collaborative network funded in 2001 by the National Institutes of Health (NIH) to conduct biological, behavioral, and clinical research in HIV-infected and HIV-at-risk adolescents, ages 12 through 24 years. In cities across the U.S. and in Puerto Rico, 15 clinical research sites at a variety of university-based medical institutions were funded to implement studies of at-risk adolescents. One such study, Connect to Protect^®^ (C2P): Partnerships for Youth Prevention Interventions, is an ATN initiative with three phases. A detailed description of C2P's methods was published previously (Ziff et al., 2006).

The protocol procedures and human subject protections information for the first two completed C2P project phases are presented below and in Table 1. The same protocols and data collection forms were submitted to each local ethics committee at the 15 sites. Protocol procedures are defined as having either an indirect or direct community focus. Though some of the C2P project's research is conducted with individuals, it has an *indirect community focus* in that its purpose is to collect information on the health of populations in particular settings in local communities. However, the majority of C2P project research is conducted with population- or community-level data. Research having a *direct community focus* refers to the community as the unit of investigation. The terms, direct and indirect community focus, will be used when we examine our findings regarding the various ethics committees' concerns about protecting individuals and communities (Objective 2).

### Phase I C2P Protocol Overview

The community is the direct focus of Phase I protocol activities. Ethics committee approval for Phase I was completed by the 15 participating research sites between January and April 2003 and consisted of three outcomes: (1) generating a youth HIV/AIDS epidemiological profile for each urban area in which an ATN/C2P site is located; (2) using the profiles to determine, within each city, geographic areas where youth morbidity and mortality cluster (i.e., high-risk areas) and a youth subpopulation on which to focus community mobilization efforts; and (3) creating official partnerships with agencies that can reach the designated youth population and that can serve the identified high-risk geographic areas. The *first outcome* was reached by mapping available data from a variety of sources such as public health departments and the Centers for Disease Control and Prevention using geographical information systems. The *second outcome* used these profiles to select a specific geographic area and population of focus at each site. The *third outcome* employed an initial, in-depth survey to determine potential community partners and create a youth service directory. In addition, an internal assessment of the functioning of the C2P multi-site project was conducted.

### Phase II C2P Protocol Overview

#### Study Procedures

The community was both the direct and indirect focus of Phase II protocol activities. Ethics committee approval for Phase II was completed by the 15 sites between November 2003 and August 2005. Since each site needed to complete the Phase I research activities prior to initiating Phase II, the ethics committee submission process was not simultaneous for the sites. The Phase II activities designated specific venues such as parks and clubs within the high-risk areas where 12- to 24-year-old youth spend time. HIV risk behaviors, social networking patterns, and HIV prevalence among youth at these venues were assessed. Initially, HIV-infected youth in treatment/care were interviewed using Audio Computer-Assisted Self-Interviewing (ACASI) technology to reveal possible venues where youth at high risk for acquiring the disease may be found. The list of possible venues nominated by HIV-infected youth was narrowed, and additional data were gathered on three to five venues per site by administering a brief venue interview to individuals who were 12 to 24 years old. The brief venue interview contained 5 questions about demographics, hypothetical availability, and willingness to complete a one-hour interview. To contextualize venue findings in the high-risk areas, ethnographic methods were used to collect additional data on the physical environment at the brief venue interview locations. Using the brief venue interview and ethnographic data, two or three venues were selected for HIV serosurvey administration to 12- to 24-year-old youth. The HIV sero-survey consisted of two components: an anonymous interview using ACASI technology and an anonymous HIV antibody assay. During protocol planning and implementation, the researcher-community partners established in Phase I discussed C2P findings and shared input on recruitment venues, strategies, safety, and other relevant topics at a series of meetings called working groups. Because partnerships with the community are at the core of the C2P and its success, these relationships were also evaluated throughout Phase II.

### Human Subject Protections

Since the purpose of the brief venue interviews was only to confirm the availability and willingness of age-eligible candidates at the venue for the planned HIV sero-survey, a shortened one-page consent form was used. The ACASI survey was administered to the HIV-infected adolescents in the clinic and to adolescents at selected venues. After the study was explained, the subject's understanding of the research was evaluated by a series of questions, and verbal informed consent was obtained. The survey was conducted anonymously and the ACASI technology immediately encrypted the data as soon as the respondent exited from the ACASI program. Questions in the ACASI were read aloud to the participants through a headset, as they appeared on the screen. This avoided the questions from being overheard. In order to maintain subject anonymity, only a coded number identified the oral (antibody assay) laboratory specimens. No identifying information was collected. To ensure extra protections during the oral sample collection procedure, each site was required to develop both a safety plan for conducting field research in their local community and a plan to provide or link HIV counseling and testing services to the study locations. Staff conducting the ACASI survey were separate from the HIV voluntary counseling and testing staff.

Due to the sensitive nature of the study, a waiver of parental permission (under U. S. 45 CFR § 46.408 (c)) for youth to participate in the ACASI survey and provide an oral specimen and a waiver of signed consent (45 CFR § 46.117 (c) (1) and (2)) were requested to maintain the anonymity of the ACASI survey and fully protect the privacy of the volunteer subjects. All sites received the waiver of signed consent, and 14 out of 15 sites were granted the parental permission waiver. The local site that was not granted a parental permission waiver, based on interpretation of state law, limited study recruitment to 18- to 24-year-olds during study implementation. The ethnographic activity was considered exempt under U.S. 45 CFR 46.101(b)(2) since it consisted of observation of public behavior at all sites. In addition, the proposed internal evaluation of the quality and structure of relationships was considered exempt under U.S. 45 CFR §46.101(b) (5) (i) by all sites. A Certificate of Confidentiality from the U.S. Department of Health and Human Services (DHHS) was acquired, even though the applicability is not clear since no identifying data were collected in this study. In addition, a waiver for the authorization for the “use and disclosure” of the collected Public Health Information under the Privacy Rule (U.S. 45 CFR Parts 160 and 164) was obtained. This waiver was necessary because the locations of recruitment venues named by participants cannot be removed since this information is related to the primary purpose of the study.

### Community Protection Concepts

The concept of community is vital to CBPR and other types of research that aim to improve population outcomes and strengthen communities by collaboration and engagement (Israel, 2002). This post-study evaluation considered how to ensure that human subject protections extend beyond the individual-level to protect communities when they are both directly and indirectly affected by research. As a multi-level project, the ATN's C2P research protocol required protections for the individual study participants as well as the communities that were engaged in this research endeavor. Four key community protection concepts integral to the protocol's approach are summarized as follows.

First, risk of harm to the group is different than risk of harm to the individual. Introducing research activities within any community carries not only the potential to harm the participating individuals but also the potential to negatively impact the community as a whole. C2P protocol procedures were devised to reduce the risk of both direct group harm to the community as well as indirect negative impacts from the individual-level research conducted in community settings. These included an extensive community venue selection process, a detailed community recruitment plan, and a community safety plan. Such protection procedures helped to ensure that the C2P researchers left the recruitment venues as they were found and free of any negative consequences from having conducted research activities. See Trickett and Levin (1990) and Christians (2000) for further discussion of ways of addressing unintended consequences of research and primary prevention activities for communities. Second, the manner in which the study staff interacts with community members in public areas such as parks and clubs is different from interacting solely with individuals in structured research settings. In this case, the controversial nature of HIV warrants extra care so as not to unwittingly stigmatize members of the community or an entire community itself. Protocol guidance for this particular study was developed for: (1) approaching subjects in their community context, (2) managing subject recruitment and enrollment in the community setting, (3) debriefing and referring subjects for clinical and social services in community settings, (4) interacting safely in the community, (5) training research staff, and (6) communicating with community partners throughout the implementation process. Sites were also encouraged to collaborate with local community-based organizations, employ local community members as staff, and incorporate community feedback from community partners, youth, and key informants into their research implementation to ensure that it was community-sensitive, culturally competent, and youth-friendly To reduce stigma and sterotyping of sub-populations, other research groups have recommended structured formal communication through the development of community advisory boards and the inclusion of community members on study staff for community-based research focused on disease prevention (Khanlou & Peter, 2005).

Third, communities impart a certain level of social comfort, or sense of place and well-being. This is evident when groups of people congregate for a common purpose and in the nature of public places themselves, and needs to be recognized during research preparation and implementation. Approaching vulnerable subjects such as at-risk adolescents in these settings considered by the youth to be their “hangouts” raises potiental justice concerns; minimizing the burden and negative impacts of these research activities for venue staff and patrons in this community space is important. Ensuring the privacy and confidentiality of the community and the individual subjects in these communal areas is paramount. The development and implementation of procedures to protect the community's privacy, as in the principle of respect for persons, requires careful strategizing. In this study, protocol procedures relied on three important measures including a data-encrypted ACASI computer interview, a detailed data management plan, and a community safety plan. Though these procedures are routinely used to protect an individual research participants' privacy, they also ensure that research implementation has only a limited impact on the community's functioning and space.

Lastly, communities need to be provided an opportunity to participate in and review research activities that affect the group. The disciplines of psychology, anthropology, and sociology have placed a high value on establishing collaborations between researchers and community members when developing and conducting research activities (Trickett, 1998; Fawcett, 1991). Structured community groups can be used for this purpose and the community members invited to participate need careful training and involvement in all stages of the research process (Oakes, 2002). The C2P project developed a variety of community partnerships in Phase I with community agencies. Partnering agencies, youth and other community members were invited to attend working group meetings as part of the Phase II protocol. Partnering agency responsibilities varied based on interest and commitment ranging from playing a substantial function in research activities to an advisory role bolstering community ownership and providing feedback Working groups contained a maximum of 15 participants and provided an opportunity for the community members to discuss general issues such as trust/distrust and ownership, to review protocol materials and implementation, give guidance on selection of community venues and safety issues, and provide feedback on community data collection.

## Method

### Data Collection Procedures

Between February and May 2006, a post-study evaluation was conducted to identify the local ethics committee concerns raised during the common CPBR protocol review process at 15 sites nationally. Correspondence and memos between clinical research sites and their ethics committees during the Phase II protocol submission process were collected from the 15 sites and analyzed. Since the review of these public documents was not considered human subjects research, it was determined that an additional local ethics committee review for this post-study evaluation was not warranted.

To carry out this research, three site directors abstracted concerns identified by the local ethics committees, documented resolutions implemented by each research site, and specified the original source document that contained the information. These data were entered into an electronic spreadsheet for data analysis. Five additional variables were also coded from the derived data. The resolution of each ethics committee concern was classified as either editorial (e.g., a clarification was requested) or substantive (e.g., justification of a study procedure was required). Each ethics committee concern and its resolution at the site were categorized according to its source (e.g., the protocol, consent form, data forms, or Health Insurance Portability and Accountability Act [HIPAA]). In addition, the three site directors applied a *who, what,* and *ethics* code to each ethics committee concern and site resolution based on a standardized data definition coding sheet during the data abstraction process. A *who* code denoted the study participant type involved in the particular ethics committee concern or site resolution. The five categories included an HIV+ subject, an HIV– subject, a subject <18 years of age, study staff, and community. A *what* code cataloged what research component the ethics committee concern or site resolution was involved. This variable comprised seven categories: HIV testing services, HIV+ ACASI administration, HIV– sero-survey administration, community research partnership interviews, brief venue interviews, and ethnographic surveys. Lastly, the ethics committee concern/site resolution was given an *ethics* code that detailed whether the identified issue was related to an untoward effect or one of the three main ethical principles detailed in the Belmont Report (National Commission for Protection of Human Subjects of Biomedical and Behavioral Research, 1979): respect for persons, beneficence, and justice. *Respect for persons* means respecting the autonomous individual's choice while protecting individuals who are incapacitated or immature. *Beneficence* refers to minimizing harm and maximizing benefits. *Justice* refers to the fairness of research subject recruitment and of the distribution of benefits and burdens of research. In addition, an untoward effect refers to a negative effect of the study that was not originally anticipated. To ensure inter-observer reliability, two out of the three site directors abstracted and coded each site's data. If an abstraction or data coding discrepancy occurred between the two site coordinators, the discrepancy was discussed and resolved by the entire study team. All ethics committee concerns/site resolutions could be classified in more than one category each for the information source and the *who, what,* and *ethics* coded variables.

## Results

Since the Phase I protocol was determined to be exempt at all 15 sites in accordance with U.S. 45 CFR § 46.101 (b) (4) and U. S. 45 CFR § 46.101(b) (5), it was not included in the post-study evaluation. For the Phase II protocol, 11 ethics committees opted for a review of the study protocol by the entire board and 4 others determined that the local research sites met the requirements for an expedited review requiring only the ethics committee chair or one or more ethics committee members designated by the chair to conduct the review of the research protocol.

Eighty-two ethics committee concerns with accompanying site resolutions were identified by 13 of the 15 participating research sites for an average of 6.3 concerns per site. Two ethics committees did not report any substantial concerns. The concerns identified by the local ethics committees were related to the consent forms (35%), protocol text (49%), data collection forms (17%), and HIPAA compliance (6%) and were reflective of traditional individual level intervention research. Approximately 12% of the concerns were considered editorial in nature; concerns that were considered substantive (*n* = 72) are presented in Table 2. The majority of substantive concerns were related to HIV– youth subjects (71%) and implementation issues surrounding the ACASI survey administration and brief venues interviews (58%).

Generally, the concerns that were raised involved individual-level subjects; only 17% of the concerns dealt with either direct or indirect community issues related to the ethical principles of beneficence (*n* = 5), justice (*n* = 6) or and/or respect for persons (*n* = 6). Specifically, the ethics committees were concerned with the evaluation of the partnerships with community members (e.g., direct community focus) and issues related to the protocol's research framework including the use of social venues in the high-risk geographic areas as recruitment sites, obtaining letters of agreement from gatekeepers of these recruitment venues and the notification of community leaders when research at the venues was being conducted (e.g., indirect community focus).

Table 3 describes the ethical classifications for the ethics committee concerns. Respect for persons was cited most often (57%) with the majority of these concerns related to informed consent documents. Forty-four percent of concerns were classified as beneficence, of which minimization of research risk participation and experimental design issues were reported most frequently. Justice concerns (19%) predominately related to participant recruitment issues ethics committee concerns related to preventing untoward effects were reported least often (6%).

## Best Practices

Sites learned three key lessons that could be applied as best practices to assist other researchers and communities in planning and ethics committees in reviewing community-based participatory research.

First, some ethics committees generalized the ethical principle of individual-level beneficence to the community. This illustrates that although current regulatory language does not address the *community* as a research subject, ethics committees considered it feasible to consider the regulations at hand and apply them as they saw appropriate. After several ethics committees examined the study's experimental design and the potential risks and benefits of conducting social network research in local communities, they requested modifications to their site's protocol procedures. In one case, notification was required to be given to community leaders affiliated with each of the social venues being used as recruitment sites. In another case, letters of agreement were required from the gatekeepers of the social venues in the community detailing their permission for research to be conducted on their premises. Researchers may want to include a community leader notification practice or social venue letter of agreement process when developing community-related research activities. When reviewing community-level research protocols, ethics committees should consider the application of beneficence widely so as to determine the appropriateness and utility of these and other related actions.

Secondly, various ethics committees took into consideration how the study staff would interact with the community during research implementation. Most of these concerns either focused on applying the principle of respect for persons to the community-level or focused on community recruitment issues when justice principles were involved. Many concerns related to ensuring the appropriateness of community-researcher interactions during the recruitment, screening, or consenting process. The concerns included the appropriate use of community language during recruitment, approaching youth in a respectful way when they were in community settings, and ensuring that community-appropriate language was used in screening and consent forms. Based on this case study, it is recommended that community researchers include in their protocol detailed research staff training and community experience description, and specific language or recruitment procedures, with special attention to approach and exit, for community fieldwork.

Lastly, several ethics committees' concerns focused on ensuring privacy and confidentiality within the community, and on ensuring that community activities continued normally and did not impact the conduct of the research or vice versa. Mostly, these concerns were about recruitment venues: that they maximized privacy and minimized community impact compared to alternative possible venues, ensured that recruitment venues and corresponding research activity schedules were convenient for youth compared to other locations and times, and ascertained that confidentiality and privacy would not be inadvertently diminished due to the recruitment location's social setting. We recommend that researchers include additional community privacy measures and a recruitment venue comparison section when developing their community research methods.

### Limitations

This study has limited generalizability since only 15 U.S. clinical research sites affiliated with a national research network implemented this protocol. In addition, since this study reviewed local level data, there was considerable variation in the ethical concerns raised by the ethics committees as well as the manner in which concerns were resolved at each site. This made it difficult to ensure that all study implications were appropriately identified. Lastly, this was a post-study evaluation that aimed to identify the ethics committee concerns of a common CBPR protocol at 15 sites to generate best practices for researchers and ethics committee reviewers and additional research questions. This type of research is post-hoc in nature and is not able to empirically test hypotheses.

## Research Agenda

Our experience developing and implementing a national community-based participatory research project emphasizes the need for ethics research focused on community concerns. We recommend the following questions as impetus for future research:

1. When does a group, such as a community, become worthy of ethical consideration?
2. How can community rights be protected in research? Are these rights dependent on the community being a direct or indirect focus of the research?
3. How can the protection of community rights be monitored over time?
4. Who consents for the community?

## Educational Implications

Since there are no regulations in the U.S. Federal Policy for the Protection of Human Subjects for conducting community-based research, investigators, study staff, and ethics committees need to carefully consider the protection of communities. We recommend that each institution require additional training related to community-level ethical and safety issues for both ethics committee members and research staff to improve community-level protections in research.

## Conclusions

Human subject protection procedures were originally designed to protect individuals participating in biomedical research. The application of the ethical principles of respect for persons, beneficence, and justice to community-level research poses unique issues that are not encountered in individual-level research. This paper described how a national community-based participatory research initiative dealt with these issues and the common concerns that local institutional review boards had when evaluating potiential risks. This paper also shared best practices based on the lessons learned both by the researchers and the review boards since there are currently no standard ethical guidelines for public health research with the community as the subject. Since there are no regulations in the U.S. Federal Policy for the Protection of Human Subjects, and probably for all national regulations of human research, for conducting community-based research, the burden falls on investigators and study staff to consider the protection of the community during study activities. Investigators and study staff recruiting communities, as participants should ensure that human protection guidelines are addressed during protocol development not only at the individual-level but at the community-level as well. In the meantime, recommendations to improve community-level protections in research include providing additional training related to community-level ethical and safety issues for both ethics committee members and research staff, adding researchers with community-level experience to local ethics committee boards to review multi-level research protocols and build community capacity, and establishing a group of community members to advise the researchers throughout the planning and implementation of the study.

## Figures and Tables

**Table 1:** Research Activities and Human Subject Protection Information by Protocol Phase.

Research Activity	Research Method	Target Population	Exemption Requested	Consent Information	Community Focus
**Phase I Protocol**					
Epidemiological Profile	Public use data; Secondary data analysis	Community	Yes	None	Direct
Area of Focus Determination	Public use data; Secondary data analysis	Community	Yes	None	Direct
Population of Focus Determination	Public use data; Secondary data analysis	Community	Yes	None	Direct
Partnership Development	Initial and In-depth Interview surveys	Community	Yes	None	Direct
Internal Organization Functioning	Survey and qualitative Interviews	Community; study staff	Yes	None	Direct
**Phase II Protocol**					
HIV+ ACASI	Computerized survey	HIV+ youth	No	Consent required; parental waiver; signed consent waiver	Indirect
Brief Venue Interview	Brief five-question interview	HIV− youth	No	Consent required; parental waiver; signed consent waiver	Indirect
Ethnographic Survey	Ethnographic survey	Community	Yes	None	Direct
HIV–ACASI	Computerized survey; anonymous HIV test	HIV− youth	No	Consent required; parental waiver;	Indirect
Working Groups	Survey and qualitative interviews	Community	Yes	signed consent waiver None	Direct
Internal Organization Functioning	Community researcher partnership interview; other surveys and qualitative interviews; survey and qualitative interviews	Community; study staff	Yes	None^1^	Direct

**ACASI:** Audio Computer-Assisted Self-Interviewing technology

1 Although the ethics committees were asked to exempt these interviews from research oversight, permission scripts were created to document that all interviewees were informed of the audio taping in the same manner, to allow for the interviewees to opt-out and to provide documentation of the interviewees acceptance of the audio taping.

**Table 2:** Frequencies and Percentages of Ethics Committee Concern by Content Type.

	Ethics Committee Concern			Total	
	
	Consent	Protocol	Forms	Frequency	Percentage
**Content Type: Who**					
HIV+ Youth	11	8	5	24	33%
HIV− Youth	18	24	10	51	71%
Youth <18 years	4	1	0	4	6%
Study Staff	2	5	0	10	14%
Community	4	8	0	12	17%
**Content Type: What**					
CRPI	2	1	0	3	4%
HIV+ ACASI	15	7	5	25	35%
HIV− ACASI/BVI	29	40	14	45	58%
HIV Testing	1	7	0	8	11%

**CRPI:** Community Research Partnership Interview, **ACASI:** Audio Computer-Assisted Self-Interviewing technology, **BVI:** Brief Venue Interview

**Note:** An ethics committee concern could affect more than one content type resulting in a total greater than the overall frequency.

**Table 3:** Frequencies and Percentages of Ethics Committee Concerns by Ethical Classifications.

Ethical Classification	EthicsCommittee Concern			Total	
	
	Consent	Protocol	Forms	Frequency	Percentage
**Justice**	**3**	**12**	**2**	**14**	**19%**
Requires attention to participant recruitment	2	10	0	11	15%
Inclusion and exclusion criteria for selecting participants	1	2	2	3	4%
**Beneficence**	**2**	**18**	**10**	**30**	**42%**
Requires an examination of the experimental design	0	6	5	11	15%
Minimization of the risks of research participation	2	10	3	15	21%
Qualifications of the principal investigator to conduct the study	0	2	2	4	6%
**Respect for Persons**	**24**	**15**	**5**	**41**	**57%**
Requires attention to informed consent information, comprehension, and voluntariness	18	4	0	22	31%
Surrogate permission	1	1	0	1	1%
Maximization of choice	2	1	1	3	4%
Protection of privacy and confidentiality	0	5	4	8	11%
Protection of vulnerable population	3	4	0	7	10%
**Untoward Effects**	**0**	**4**	**0**	**4**	**6%**
Study staff safety	0	4	0	4	6%

**Note:** A single ethics committee concern may have multiple ethical labels, resulting in table totals in excess of the seventy-two substantive ethics committee concerns. In addition, one ethics committee concern could affect more than one content type resulting in a total greater than the overall frequency.
